# Supplementary material for: Preliminary Biological Activity Screening of Plectranthus spp. Extracts for the Search of Anticancer Lead Molecules
Source: Pharmaceuticals (Basel). 2021 Apr 23;14(5):402. doi: 10.3390/ph14050402 (PMC8146581; doi:10.3390/ph14050402)
Supplement: Supplementary file 1 [file pharmaceuticals-14-00402-s001.zip › pharmaceuticals-1193181-supplementary.pdf]

pharmaceuticals

Article

Preliminary biological activity screening of *Plectranthus* spp. extracts for the search of anticancer lead molecules

Supporting Information

Figure S1: ^{1}H-NMR data information for 7α-acetoxy-6β-hydroxyroyleanone isolated from *P. hadiensis*

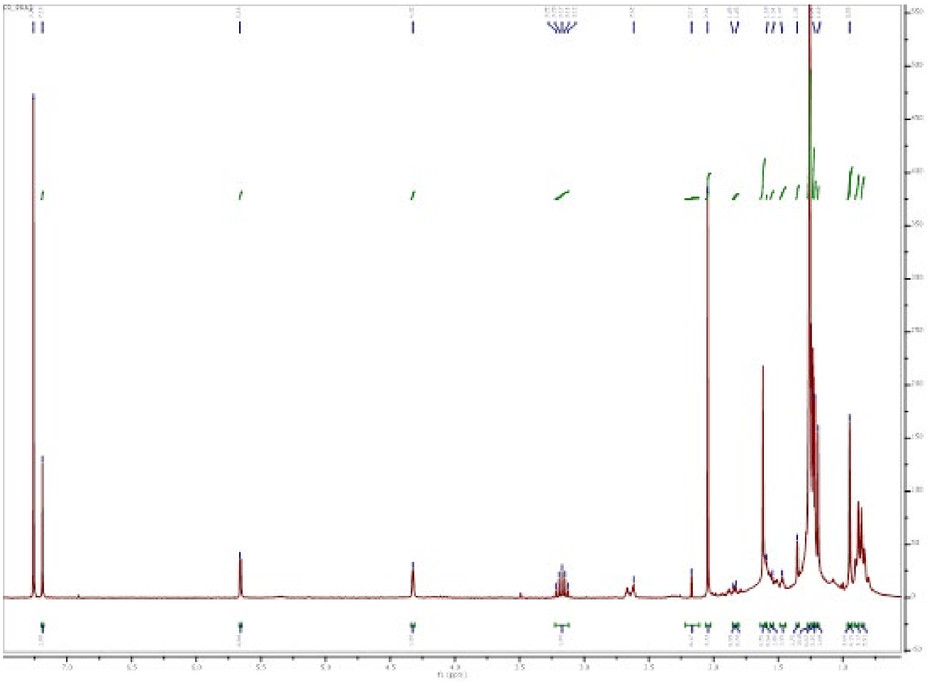

Table S1: NMR spectroscopy data characterization, ^{1}H NMR (300 MHz, $CDCl_3$), ^{13}C (75 MHz, $CDCl_3$

Position	δc types	δH (J =Hz)	HMBC
1	38.46	2.62, m (2.6)	3
2	21.69	1.83 2β, dd (13.5, 3.7)<br>1.54 2α, d (3.6)	
3	42.43	1.47, d (1.3)	1, 2, 4
4	33.84		
**5**	49.92	1.35, s	3
6	67.07	4.32, 6-H,d(1.7)<br>2.17, 6-OH, s	5, 7, 8
7	68.89	5.66, d (2.1)	6, 5
8	136.99		
9	150.04		

10	38.85		
11	182.99		
12	150.05	7.19, s	11, 13
13	124.76		
14	185.89		
15	24.35	3.17, sept (7.5)	13, 14, 16, 17
16	20.01	1.20 ,d (7.1)	
17	19.88	1.19, d (7.1)	
18	33.78	0.95, s	3
19	23.85	1.2, s	
20	21.54	1.59, s	11
7α-$\underline{C}OCH_3$	169.70		
7α-$CO\underline{C}H_3$	21.06	2.04, s	7α-$\underline{C}OCH_3$

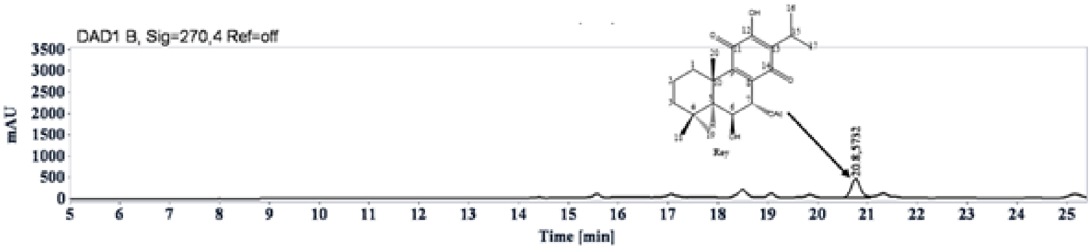

Figure S2: HPLC representative chromatograms (270 nm) of *P. hadiensis* leaves showing the major compound, 7α-Acetoxy-6β-hydroxyroyleanone

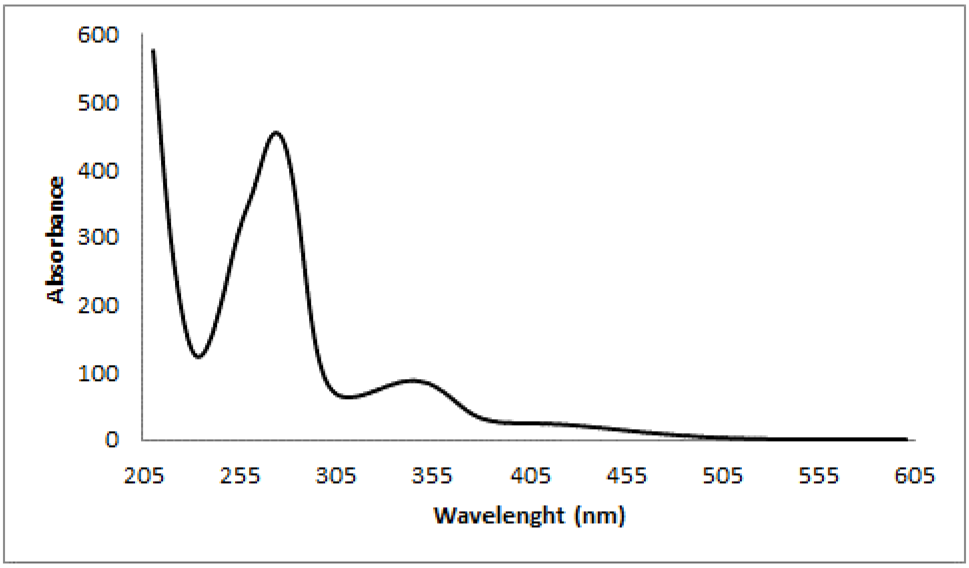

Figure S3: UV spectrum of 7α-Acetoxy-6β-hydroxyroyleanone

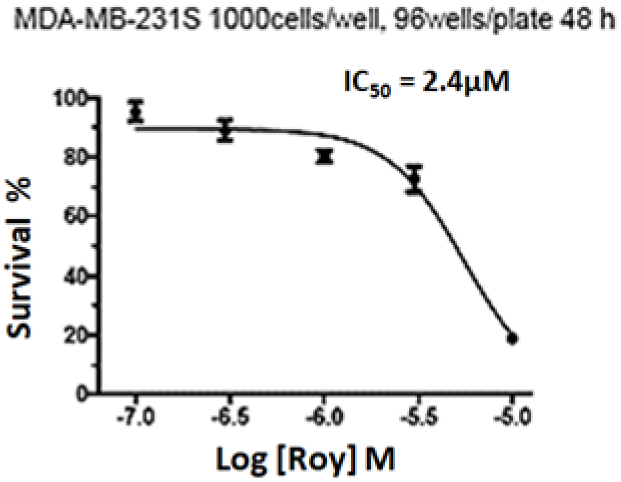

Figure S4: Concentration-response curves (IC_{50} μM) for Roy
